# Improved diagnosis of pulmonary embolism causing cardiac arrest by combined endobronchial ultrasound and echocardiography

**DOI:** 10.1186/s12947-020-00208-z

**Published:** 2020-07-06

**Authors:** Pietro Bertini, Alessandro Ribechini, Fabio Guarracino

**Affiliations:** 1 Department of Anaesthesia and Critical Care, Cardiothoracic and Vascular Anaesthesia and Intensive Care Medicine, Azienda Ospedaliero Universitaria Pisana, Via Paradisa 2, 56124 Pisa, Italy; 2 Thoracic Endoscopy Unit, Azienda Ospedaliero Universitaria Pisana, Pisa, Italy

**Keywords:** EBUS, Cardiac arrest, Pulmonary embolism, Echocardiography, Ultrasound

## Abstract

**Background:**

Pulmonary embolism (PE) is a life-threatening disease difficult to diagnose and manage in severe hemodynamic unstable patients. Transoesophageal echocardiography (TEE) is considered useful to improve diagnosis, but such approach has physical limitations for the interposition of the airways preventing the clear assessment of the left pulmonary artery. Endobronchial ultrasound (EBUS), a recently developed technique carried out using a modified bronchoscope having a small ultrasound convex probe at the tip allowing to perform ultrasonography examination of the mediastinum, can extensively visualize the pulmonary arteries on both sides.

**Case presentation:**

We present the first use of EBUS to rapidly diagnose and subsequently treat a 64 years old woman with history of lateral amyotrophic sclerosis admitted to the intensive care unit (ICU) for severe dyspnoea and rapidly experiencing a cardiac arrest.

**Conclusions:**

Combined bedside EBUS and echocardiography allowed to rapidly diagnose the cause of cardiac arrest and avoid risks related to transferring the critical patient to the radiology department.

## Background

Pulmonary embolism (PE) is a life-threatening disease difficult to suspect and diagnose. The gold standard to discover and assess for PE severity is angiographic CT scan [1], but in patients with severe hemodynamic instability the transfer to the radiology suite can be dangerous or impossible. In such cases, echocardiography has been demonstrated useful to detect right intra-atrial or intra-ventricular masses resembling thrombi and to confirm the suspect of massive PE in patient with cardiac arrest or peri-arrest arrhythmias with no evidence of a cardiogenic cause [1]. Unfortunately, transthoracic echocardiography (TTE) can be difficult to perform in peri-arrest during resuscitative manoeuvres, therefore, transesophageal echocardiography (TEE) can be considered to improve diagnosis allowing to visualize the main stem of the pulmonary artery (PA) as well as the uppermost part of the right PA [2]. Such approach though, has physical limitations for the interposition of the airways which prevent from a clear assessment of the left pulmonary branch.

**Fig. 1:**
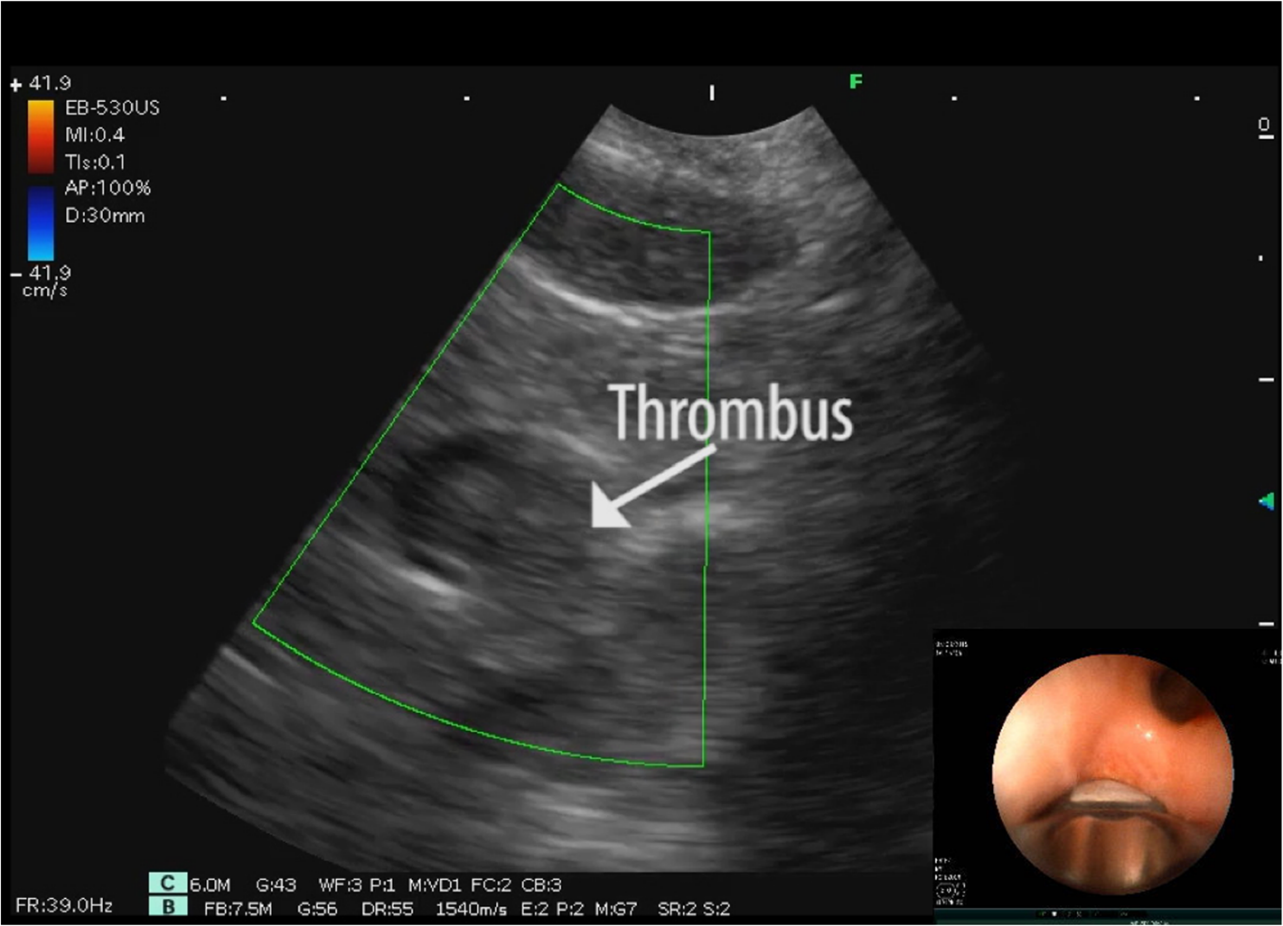
EBUS image showing the thrombus in the distal branch of the left pulmonary artery, with the tip of the probe in the left lower lobe bronchus (left panel)

Endobronchial ultrasound (EBUS) is a technique recently developed by thoracic endoscopists for real-time ultrasound-guided needle biopsy of mediastinal lymph nodes for staging lung cancer [3]. It is carried out using a modified bronchoscope having a small ultrasound convex probe at the tip allowing to perform ultrasonography examination of the mediastinum by applying gentle pressure to the trachea or the bronchial inner surface (Fig. 1). EBUS allows extensive visualization of both left and right pulmonary artery branches. We here report a case of cardiac arrest in which EBUS exam rapidly diagnosed PE as the cause of the hemodynamic collapse.

## Case presentation

A 64 years old woman with history of lateral amyotrophic sclerosis, was admitted to the intensive care unit (ICU) for severe dyspnoea and shortness of breath. Soon after instrumentation with a radial artery catheter for hemodynamic monitoring, she developed severe hypotension and eventually cardiac arrest. She was resuscitated and a periarrest TTE was performed showing severe right ventricular failure (RVF) and dilatation. Suspecting PE, TEE was performed and no signs of emboli were seen in the main stem of the pulmonary artery nor in the upper part of the right pulmonary branch, although RVF was confirmed. As expected, the left branch of the pulmonary artery was impossible to visualize, but the patient was still in severe hemodynamic shock to be safely transferred for a CT scan. In fact, she suffered a second cardiac arrest requiring resuscitation manoeuvres again. In order to confirm the diagnostic hypothesis, we decided to have an emergent bedside EBUS executed. The exam was carried out in a few minutes confirming the absence of thrombi in the main PA and in the right PA (Clip 1), but allowing to see coagulated masses in the left PA (Clip 2). Based on the severe clinical situation, the TTE and TEE exam showing RV overload and the EBUS images showing the presence of thrombi in the pulmonary circulation, we decided to treat the patient with intra-venous thrombolysis to address the embolised material. Following the treatment, the patient quickly recovered hemodynamically. EBUS was carried out as follow up over the next few days demonstrating almost full thrombus resolution (Clip 3).

## Discussion and conclusions

The case reported displays once again the importance of ultrasound imaging in the bedside diagnosis of cardiac arrest etiology; although ultrasound is a powerful tool to detect several conditions responsible for hemodynamic collapse, and rapidly reversible, such as an increase in pericardial pressure, massive hemothorax, tension pneumothorax or a gross valvular insufficiency, echocardiography even in the transesophageal approach is only able to detect a massive pulmonary embolism when thrombii are visible in the right heart or in the pulmonary arterial tree or diagnose an acute right ventricular failure suspicious of a sudden obstruction of its output.

Unfortunately, more distal and undetectable embolised material can also be responsible of hemodynamic alteration and eventually cardiac arrest as for our case. In such situation, we recognised bronchial ultrasound as a powerful tool to rapidly diagnose pulmonary embolism as the aetiology of the cardiac arrest and guide the clinical team in the resuscitation efforts. Our experience in a cardiac arrest situation differs from previous reports on EBUS in that in elective patients PE was reported as an incidental finding [4], and in an acute case [5] with severe sepsis related hypotension to exclude PE, despite the title suggesting a diagnosis of acute PE.

In our experience EBUS offered exceptional imaging quality for the diagnosis of PE being able to clearly visualize the distal portions of the pulmonary vasculature even in areas not amenable to TEE exploration. Moreover, it was carried out in a few minutes by a skilled thoracic endoscopist assisted by a cardiac anaesthetisiologist expert in cardiovascular ultrasound, and allowed to spare the patient greater risks associated with being transferred to another department for a CT scan.

As targeting therapeutics using bedside focused approach by ultrasound is expanding, offering a tailored approach to the critically ill [6] also thanks to point-of-care machines, future application of new modalities of cardiovascular ultrasound in critical care and anaesthesia, like in our endobronchial approach, have to be explored. Finally, although this approach seems to be reasonable, beneficial effects in terms of outcome need to be evaluated in perspective larger studies.

## Supplementary information

**Additional file 1 Clip. 1.** EBUS clip of the right pulmonary artery (RPA). Central venous catheter (CVC) is visible in the lower part of the screen.

**Additional file 2 Clip. 2.** Colour Doppler Ultrasound of a distal branch of the left pulmonary artery. A thrombus is indicated by the white arrow.

**Additional file 3 Clip. 3.** Follow up EBUS exam of the left pulmonary artery. Residual thrombotic material in the left pulmonary artery – white arrows.

## Data Availability

Not applicable.

## Abbreviations

TEE: Transesophageal echocardiography
TTE: Transthoracic echocardiography
EBUS: Endobronchial ultrasound
ICU: Intensive care unit
PE: Pulmonary embolism
CT: Computed tomography
PA: Pulmonary artery
RVF: Right ventricular failure

## References

[1] Konstantinides SV, Torbicki A, Agnelli G (2014). 2014 ESC guidelines on the diagnosis and management of acute pulmonary embolism. Eur Heart J.

[2] Jelic T, Baimel M, Chenkin J (2017). Bedside identification of massive pulmonary embolism with point-of-care transesophageal echocardiography. J Emerg Med.

[3] Colella S, Scarlata S, Bonifazi M (2018). Biopsy needles for mediastinal lymph node sampling by endosonography: current knowledge and future perspectives. J Thorac Dis.

[4] Sariaydin M, Gunay S, Gunay E, Sarinc US (2016). Endobronchial ultrasound: an unusual diagnostic tool for pulmonary embolism. Am J Emerg Med.

[5] Channick CL, Channick RN (2019). Use of endobronchial ultrasound for bedside diagnosis of acute pulmonary embolism in a critically ill patient. Chest..

[6] Guarracino F, Bertini P, Pinsky MR (2018). Novel applications of bedside monitoring to plumb patient hemodynamic state and response to therapy. Minerva Anestesiol.

